# Joint Simon effect in rats: Validation across two strains and exploratory analysis of the influence of familiarity

**DOI:** 10.1371/journal.pone.0328527

**Published:** 2025-08-18

**Authors:** Noriko Katsu, Kazuo Okanoya

**Affiliations:** 1 Graduate School of Arts and Sciences, The University of Tokyo, Megruo, Tokyo, Japan; 2 Graduate School of Human Sciences, Osaka University, Suita, Osaka, Japan; 3 Advanced Comprehensive Research Organization, Teikyo University, Itabashi, Tokyo, Japan; Technion Israel Institute of Technology, ISRAEL

## Abstract

Examining the influence of others on joint actions could clarify the basal mechanisms underlying social coordination in non-human animals. The joint Simon task is used for this purpose, and some non-human animals have demonstrated the joint Simon effect. However, previous investigations have only demonstrated the effect among familiar dyads despite the potential importance of social relationships for task performance. In this study, we investigated joint Simon effects using two rat strains (Wistar and Long-Evans) that differ in appearance. We performed exploratory testing to examine whether familiarity between dyads affects the size of the joint Simon effect. We compared the joint Simon effects that emerged when subjects (N = 8) were paired with familiar cagemates to when they were paired with stranger non-cagemates. After discrimination training, the rats performed the joint Simon task with two auditory stimuli (2 and 4 kHz tones). Rats showed a significant joint Simon effect when paired with a cagemate partner, replicating earlier findings. Although rats showed a greater compatibility effect when paired with cagemates than with non-cagemates, the small sample size and repeated dyads warrant cautious interpretation. Our findings suggest that the attention of rats is drawn to adjacent partners during a joint task, even in the absence of explicit benefits. The exploratory results on familiarity suggest the possibility that perceived similarity between partners induces a larger joint Simon effect in rats.

## Introduction

Cooperation among multiple animals requires mutual monitoring to coordinate or collaborate on actions [1]. Boesch and Boesch [2], who studied cooperation in group hunting among non-human primates, defined cooperation as the behavior in which two or more individuals act together to achieve a common goal. According to the operational definitions [2], collaboration indicates that participants complement each other with different actions, while coordination means that participants align their same actions in time and space with one another. In addition to the explicit cooperative behaviors that benefit participants, an individual’s behavior may affect that of an adjacent individual simply because of their proximity. For example, non-human animals exhibit behavioral contagion [3,4], that is, involuntary transmission of behavior between individuals that is not directly related to a benefit (e.g., yawning contagion). Therefore, examining the influence of others on joint actions facilitates the identification of the basal mechanisms underlying social cognitive capacity, such as social transmission and facilitation [5,6]. In the current study, we used the experimental paradigm of the joint Simon task [7] to examine how the passive monitoring of the actions of others interferes with performance in non-human animals.

The joint Simon task is used to examine the cognitive processes of humans engaged in joint and collaborative actions [7]. This is based on the Simon task, which elicits a spatial stimulus-response compatibility effect [8]. In the Simon task, a participant engages in a two-choice discrimination task in which the spatial arrangement of the stimulus presented and the correct response key are either compatible or incompatible. For example, when the correct response for stimulus A (which has no spatial attribution, such as a visual or auditory stimulus) is to respond using the left key, a participant typically shows a more rapid reaction time and lower error when responding to stimulus A presented from the left side; that is, the stimulus and response are compatible in spatial position. In contrast, participants typically show slower reaction times and more errors if the same stimulus is presented from the right side; thus, the response is incompatible. This interference effect of incompatibility is known as the compatibility or Simon effect [8]. This effect disappears when a participant engages in one half (go/no-go task) of a Simon task divided into two half-tasks (left and right) because of the lack of a spatial code for the response in a half-task [9]. However, when two participants share two half-tasks side by side, the compatibility effect is similar to that observed when one person performs the full task alone. This phenomenon is called the joint Simon effect [7].

Two major explanations have been provided for the joint Simon effect: shared representation of the partner’s action (action co-representation) [7,10] and referential coding account [11,12]. The joint Simon task in humans can be classified as collaborative behavior, according to Boesch and Boesch [2], because the two participants share a complementary task set and co-represent the action of their partner [7]. However, the joint Simon task itself can be completed if two participants perform their own half-tasks and ignore the other half. In this case, the task may not explicitly include collaboration or coordination. Nonetheless, the joint Simon task elucidates the influence of the actions of adjacent individuals who share benefits on one’s own actions.

The former explanation by action co-representation assumes a relatively high order of social cognitive ability for non-human animals, namely, understanding the role of the partner [13]. Regarding the latter, participants referred to a partner as a reference point for their actions, and no co-representation was required [12]. It is also reported the perception of agency, and not that of the partner’s intentionality, is considered sufficient for the occurrence of the joint Simon effect [14]. Our study focused on rats, in which various examples of social coordination have been reported [15]. To the best of our knowledge, previous reports have not directly examined agency perception in rats. However, differential behavior toward conspecifics and objects (inanimate toys) has been reported in helping behaviors in rats [16] and in social preference tests in other rodents [17]. We assumed that at least some sensitivity to conspecifics that exhibit biological behaviors exists in rats.

One interesting feature of the joint Simon effect is that in humans, social relationships are positively associated with the size of the interference effect [18–20]. This indicates that social factors influence the shaping and discrimination between action and event representations [19]. The simple Simon effect for auditory stimuli has been demonstrated in several non-human species, including rats [21,22]. In addition, it is considered robust in non-human animals [23–25]. The joint Simon effect was recently demonstrated in both non-human primates [26,27] and rats [28]. One study on primates examined the effects of social networks or dyadic sociality indices on the joint Simon effect; however, no significant effects were reported [29]. These studies have shown that the joint Simon effect occurs among familiar individuals (i.e., individuals from the same social group). However, whether the joint Simon effect occurs with unfamiliar individuals has not been considered in nonhuman animals. Therefore, it would be worthwhile to compare the size of the effect between familiar and unfamiliar dyads, even if it is a preliminary examination.

The relationship between subjects in a joint action may affect the outcome in rats. For example, cooperative experiments using robotic rats demonstrate the importance of social communication between dyads [30]. Previous studies have investigated whether social behaviors such as social transmission, emotional contagion, and cooperative behavior are differentiated based on individual familiarity [31–33]. However, there is limited evidence regarding the effect of familiarity on these social behaviors, although rats can discriminate between familiar and unfamiliar individuals [34], even when based only on odor cues [35]. Furthermore, male rats form differentiated social relationships [36,37], indicating their preference for particular individuals. Although the joint Simon task includes social factors, explicit social coordination is not required. These characteristics help examine the influence of social relationships on the actions of rats from a perspective that is not based on emotions.

In this study, we aimed to examine the joint Simon effect by extending the subjects from previous studies to two strains of rats that present differences in appearance. We performed an exploratory evaluation on whether familiarity between individuals affects the occurrence of the joint Simon effect and compared the joint Simon effect on a dyad of cagemates that had lived together for a certain period with that on a dyad of non-cagemates. This exploratory result was used to seek a hypothesis on how social relationships affect joint action in rats.

## Materials and methods

### Statement of ethical approval

The experiments in the current study were conducted following the experimental implementation regulations of the University of Tokyo and ARRIVE guidelines [38] when applicable. This study was approved by the Animal Experimental Committee of the University of Tokyo Graduate School of Arts and Sciences (approval Number: 27−8).

### Subjects and housing

Male Long-Evans (n = 4) and Wistar rats (n = 4, Japan SLC, Inc., Shizuoka, Japan) were used. The two strains differ in appearance. They were housed in pairs with other individuals from one month before the experiment (temperature: 23 ± 3 °C; 12 h light/dark cycle, lights on at 8:00 am) at the University of Tokyo in Japan. The subjects were divided into two housing conditions to investigate the effects of strain differences: single-strain (Wistar pair and Long-Evans pair) and mixed-strain (two Wistar and Long-Evans pairs) conditions. Subjects in the single-strain condition had no contact with the other strain before testing. We started the experiment when the rats were three months old. Food and water were freely available before the experiments. Food was restricted to 16 g/day, except for food rewards, after the beginning of the experimental period.

### Apparatus

We used an operant box that could be divided into two chambers with a wire mesh wall (interior: 330 mm W × 300 mm D × 250 mm H; OPR-3601; O’Hara & Co., Ltd., Tokyo, Japan). The two subjects were able to contact each other through the wall. The box was then placed in a sound-attenuating chamber. Two levers and pellet dispensers were symmetrically situated on the front panel. The stimuli were 2 and 4 kHz tones played at 50–55 dB sound pressure levels (SPLs). The stimuli were presented from two speakers (5 W, 8 Ω impedance, 65 mm in diameter) connected to a power amplifier (AP20d; Fostex, Tokyo, Japan) and an audio interface (U-PHORIA UMC202; Behringer, Willich, Germany).

### Task

Following Courtiere et al. [21], the rats were trained to discriminate between two auditory stimuli. The task and training processes were essentially the same as those used in a previous study [28]. At the beginning of the trial, 2 or 4 kHz stimuli were presented for 3 s. If the rats pressed the left lever in response to a 2 kHz stimulus or the right lever in response to a 4 kHz stimulus, the trial was judged to be correct. Trials in which the rats did not press the lever for 15 s were considered omissions. Stimuli were played by both left and right speakers during the training sessions. The combination of the stimulus and the correct lever was the same for all the subjects. Two purified rodent tablets (20 mg, 5TUL; TestDiet, St. Louis, MO, USA) were provided by both the left and right dispensers for a correct response. The trial was considered an error if the rat pressed the opposite lever; a 15 s delay was inserted before the subsequent trial.

A stimulus was played by either the right or left speaker in the test session; therefore, the location of the stimulus and the required response could be either compatible or incompatible. When the 2 kHz stimulus was presented by the left speaker and the 4 kHz stimulus was presented by the right speaker, the required response was compatible (left and right lever, respectively: the compatible condition). Conversely, when the 2 kHz stimulus was delivered from the right speaker and the 4 kHz stimulus was presented from the left speaker, the required response was incompatible (incompatible condition). Other details were the same as those used in the training trials. The details of the test trial conditions are listed in Table 1.

**Table 1:** Summary of the four trial conditions used in the test sessions.

Trial condition	Stimulus	Stimulus position	Correct response	N of trials	Amplitude (dB SPL)
Compatible-L	2 kHz	L	L	25	53
Incompatible-L	2 kHz	R	L	25	54
Compatible-R	4 kHz	R	R	25	50
Incompatible-R	4 kHz	L	R	25	51

SPL, sound pressure level; L, left; R, right.

### Training process

On the first day, the rats were individually habituated to the box and underwent magazine training for 30 min. Lever-pressing behavior was shaped for four days, after which the training began. The training sessions consisted of 50 trials each at 2 kHz and 50 trials at 4 kHz. The order of the trials was pseudo-randomized according to Fellows sequences [39]. The test sessions began when the subject achieved an 85% correct response rate for both stimulus types (2 and 4 kHz) in two successive sessions.

### Task condition

**Fig 1:**
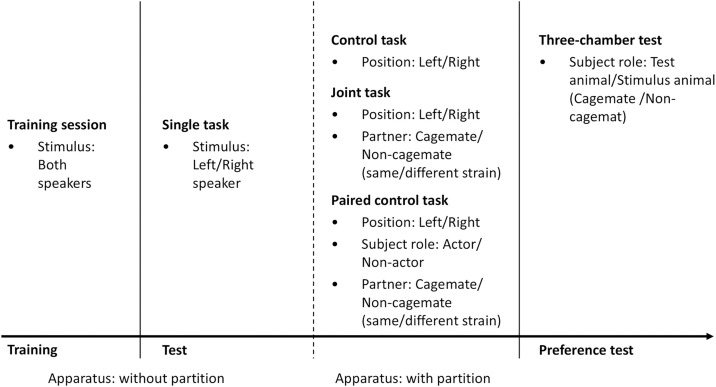
The flow chart of the experiment. The rats were subjected to the single task after training, followed by the other three tasks with a cagemate and two non-cagemate partners. After all the tests were completed, each subject participated in the three-chamber test as both a subject and a stimulus animal.

The task conditions comprised single, control, joint, and paired control tasks (Fig 1). In the single task, the subject was placed in the box alone, and test trials were performed. In the control task, the box was divided by a wire mesh wall, and the subject was placed on one side of the box. The single task was divided into right and left halves, and the subjects performed half of the task. The subject had access to either the left or right lever. Therefore, the subjects were required to perform a go/no-go task in the control task, ignoring the stimulus corresponding to the lever on the opposite side of the partition. Correct no-go responses were rewarded in this test. In the joint task, two subjects were introduced to each side of a box chamber. They performed the same go/no-go task as the control task. In the paired control task, two subjects were introduced into the box; however, only the subject located on one side of the box performed the task throughout the session, and only that subject was rewarded. A reward was given for the correct go and no-go responses. The other conditions were the same for both tasks. The two subjects habituated for 5 min before the beginning of these two tasks. Each subject performed the control task in both the left and right compartments.

We conducted an exploratory experiment to compare the joint and paired control tasks between the cagemate and non-cagemate conditions to examine the effects of familiarity, strain, and similarity in appearance between the partners. Familiarity between partners was defined when the partner was a cagemate. Each subject performed joint and paired tasks with a cagemate, non-cagemate from the same strain, and non-cagemate from the different strain.

For each subject, a single task was conducted first, followed by the other three tasks performed in random order (Fig 1). The order of the tasks, including whether the cagemate or non-cagemate condition was conducted earlier, was counterbalanced among the subjects.

### Preference test

A three-chamber test [17] was conducted to investigate whether the rats could distinguish between cagemates and unfamiliar individuals. The testing apparatus consisted of a black acrylic square arena (800 × 800 × 500 mm) divided into three compartments using transparent partitions, with two wire restraint chambers (230 × 180 × 200 mm) placed in each compartment (S1 Fig). Cagemates and non-cagemates were placed in each restraint chamber. The test animal was able to enter and exit all chambers through square openings (100 mm × 100 mm) at the center bottom of the transparent partitions. A pretest of habituation to the arena was conducted before the testing day. The subjects were placed in the center chamber and allowed to freely explore the chamber for 5 min. The test consisted of two phases: habituation (5 min) and test (5 min), with a 2 min interval between the phases. No animals were placed in the restraint chamber during habituation. The test subject was released into the center chamber as in the pretest habituation. The animal was removed from the arena after 5 min, and then a 2 min interval began. During this period, the arena was cleaned (if necessary), and two stimulus animals were placed at either end of the restraint chamber (counterbalanced). During the test phase, animals were returned to the center chamber and released for social exploration. The non-cagemate stimulus animal was an individual who performed a joint task with a testing animal of the same strain as the cagemate of the testing animal. All subjects underwent this test once as test animals and twice as stimulus animals. The order of pretest habituation and testing was counterbalanced among the cagemates.

### Data analyses

#### Compatibility effect.

Based on a previous study of the joint Simon effect in non-human animals [26], we analyzed the correct response rate. The compatibility effect indicates the size of the conflict between the location of the stimulus and the required response. We calculated the compatibility effect by dividing the correct response rates of compatible trials by those of incompatible trials. The compatibility effect was calculated based on the task and sound stimulus conditions. Go and no-go trials were included in the control and paired control tasks, and the correct rates were generally higher in the go trials than in the no-go trials. Therefore, the effect was calculated separately for go and no-go trials in the control and paired-control tasks. We excluded omission trials, trials with reaction times <130 ms (anticipated response [21]), and those with reaction times >3 s (delayed response) from our calculations.

#### Preference test.

Video recordings of the three-chamber test were analyzed using an ANY-maze (Stoelting, Wood Dale, IL, USA). The total time spent in the front area (180 × 230 mm; S1 Fig) of each restraint chamber was calculated for both the habituation and testing phases. Differences between the habituation and test phases were used for analysis to control for individual left-right bias.

#### Statistical analyses.

We conducted a Wilcoxon signed-rank test using the package ‘exactRankTests’ and linear mixed model (LMM) analyses using ‘lme4’ and ‘lmerTest’ in R version 4.0.3 [40]. The response variable in the LMM analysis was the compatibility effect. Subject ID (both models) and partner ID (the second model) were entered as random effects. The validity of the fitted models was confirmed using the package ‘DHARMa’.

First, we investigated whether the Simon effect was greater when sharing half the task or performing the full task alone than when performing the half-task alone (control and paired control conditions). The fixed effects were the task condition (four levels: single, control, joint, and paired control), sound stimulus condition (2 or 4 kHz), the strain of the subject (Wistar or Long Evans), and whether the trials were go or no-go trials.

We performed an exploratory test to determine whether familiarity affects the occurrence of the joint Simon effect. The fixed effects were the task condition (two levels: joint or paired control), familiarity between the subjects (cagemate or non-cagemate), interaction effect of these two variables, sound stimulus condition (2 or 4 kHz), strain of the subjects (Wistar or Long-Evans), strain combination between the two subjects (different or the same strain), and trial type (go or no-go trials).

We conducted a likelihood ratio test to compare the full model with the model without the effects of task conditions or interaction effects to determine the significance of the variable. When these effects were significant, pairwise comparisons were conducted for the effects among the four task types and the interaction effect between task type and familiarity using the ‘emmeans’ package.

## Results

### Comparison among task conditions

The subjects completed the training for an average of 74.5 ± 24.9 (mean ± SD) sessions. The number of sessions used in this analysis is summarized in the S1 Table. The average overall correct rate for each task was 69.3 ± 11.3% (omission rate: 4.3 ± 2.9%), 57.7 ± 6.1% (4.1 ± 2.8%), 59.3 ± 5.6% (4.0 ± 3.3%), and 57.1 ± 8.3% (5.5 ± 5.6%) for the single, control, joint, and paired control tasks, respectively.

**Fig 2:**
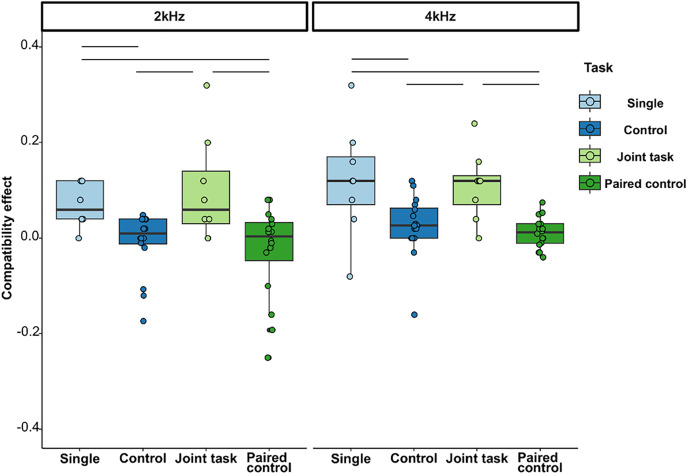
Boxplots for the compatibility effect in the single, control, joint, and paired-control conditions with two stimulus conditions. Each plot indicates the compatibility effect for go and no-go trials. The significant differences determined via multiple comparison are shown in connecting lines. Note that the Y-axis is limited to −0.40 to 0.40 for readability (full range = −1 to 1).

Fig 2 shows a comparison of the compatibility effects for each task condition. The effect of the task condition was significant (subjects, N = 8; data points, N = 96; χ^2^ = 22.361; df = 3; p < 0.0001, Table 2). A pairwise comparison revealed that the compatibility effect in the single and joint conditions was higher than that in the control and paired control conditions, indicating the occurrence of Simon and joint Simon effects (Fig 2; S2 Table). No significant differences were observed between the paired control and control conditions or between the single and joint conditions (Fig 2). We observed that the compatibility effect was higher in the 4 kHz stimulus trials than in the 2 kHz trials. The other variables were not statistically significant.

**Table 2:** Summary of the LMM of the effects of the four task conditions, stimulus type, and subject strain on the compatibility effect.

Explanatory variables		Estimate (SE)	95% CI	t	p
Intercept		−0.008 (0.020)	[-0.043, -0.001]	4.362	<0.0001
**Task**	**Single**	**0.087 (0.022)**	**[0.040, 0.113]**	**3.191**	**0.002**
	**Joint**	**0.097 (0.022)**	**[0.049, 0.127]**	**3.612**	**0.0005**
	Paired control	−0.016 (0.018)	[-0.045, 0.015]	−0.906	0.368
Strain	Wistar	0.003 (0.017)	[-0.027, 0.032]	0.181	0.858
**Stimulus**	**4 kHz**	**0.035 (0.015)**	**[0.012, 0.061]**	**2.392**	**0.019**
Trial	No-go	0.035 (0.015)	[-0.052, 0.007]	−1.252	0.214

Significant variables are shown in bold.

### Exploratory results of the effect of familiarity on the compatibility effect

The correct rates for the non-cagemate condition were 60.6 ± 20.1% for the joint task and 57.4 ± 6.2% for the paired control task. No differences were observed in the overall correct rates between the cagemate and non-cagemate conditions for the joint task (paired t-test; t (7) = 0.153, p = 0.883) or paired control task (t (7) = 0.785, p = 0.458). The relatively high deviation in the correct rate for the joint task with non-cagemates was attributed to one subject (K17). The number of sessions used in the analysis is summarized in the S3 Table.

**Fig 3:**
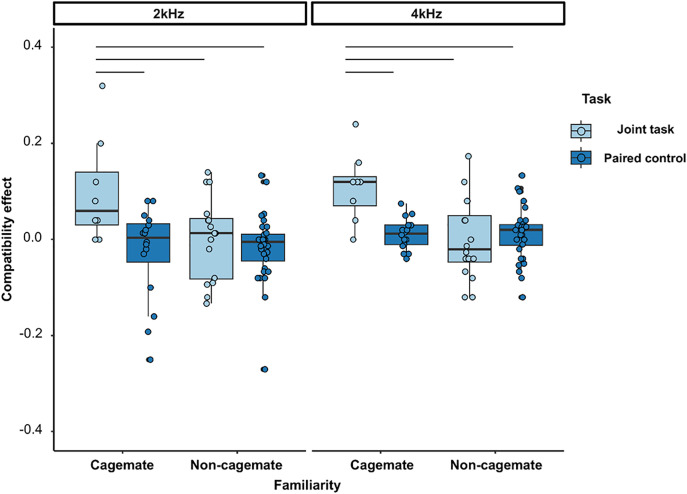
Boxplots for the compatibility effect in the joint and paired-control conditions based on familiarity (cagemate dyad: n = 4; non-cagemate dyad: n = 8). The compatibility effect in joint task conditions of the familiar dyads was significantly higher than that in the unfamiliar dyads (Table 3; S4 Table). Note that the Y-axis is limited to −.40 to.40 for readability (full range = −1 to 1).

Exploratory analysis of the compatibility effects of joint and paired control tasks by cagemate and non-cagemate dyads revealed a significant interaction between the task condition and familiarity of the dyad (LMM and the likelihood ratio test: subjects, N = 8; data points, N = 144; dyads, N = 12; χ^2^ = 16.242, df = 1, p < 0.0001; Table 3; S4 Table). We compared the simple effects of the task conditions in cagemates and non-cagemates. The compatibility effect was significantly greater in the joint task than in the paired control task in the cagemate condition (p < 0.0001), whereas no difference was observed in non-cagemates (p = 0.701; Fig 3). The compatibility effect of the joint task in the cagemate condition was greater than that in the non-cagemate condition (p < 0.0001). No significant differences were observed between the paired control tasks in the cagemate and non-cagemate conditions (p = 0.701). Other factors, such as subject strain or a combination of strains, were not significant.

**Table 3:** Summary of the exploratory LMM on the effect of the task conditions, cagemate conditions, stimulus type, and subject strain on the compatibility effect between joint and paired control tasks.

Explanatory Variables		Estimate (SE)	95% CI	t	p
Intercept		0.111 (0.021)	[0.079, 0.148]	5.391	< 0.0001
Task*Familiarity	Paired control*Unfamiliar	**0.111 (0.027)**	**[0.067, 0.160]**	**4.146**	**< 0.0001**
**Task**	**Paired control**	**−0.106 (0.023)**	**[-0.150, -0.071]**	**−4.637**	**<0.0001**
**Familiarity**	**Unfamiliar**	**−0.104 (0.022)**	**[-0.141, -0.071]**	**−4.798**	**<0.0001**
Strain	Wistar	−0.013 (0.012)	[-0.031, 0.008]	−1.079	0.282
Pair type	Same strain	−0.020 (0.012)	[-0.041, 0.000]	−1.720	0.088
Stimulus	4 kHz	0.022 (0.012)	[0.001, 0.040]	1.842	0.068
Trial	No-go	−0.013 (0.027)	[-0.037, 0.010]	−0.927	0.356

Significant variables are shown in bold.

### Preference test

**Fig 4:**
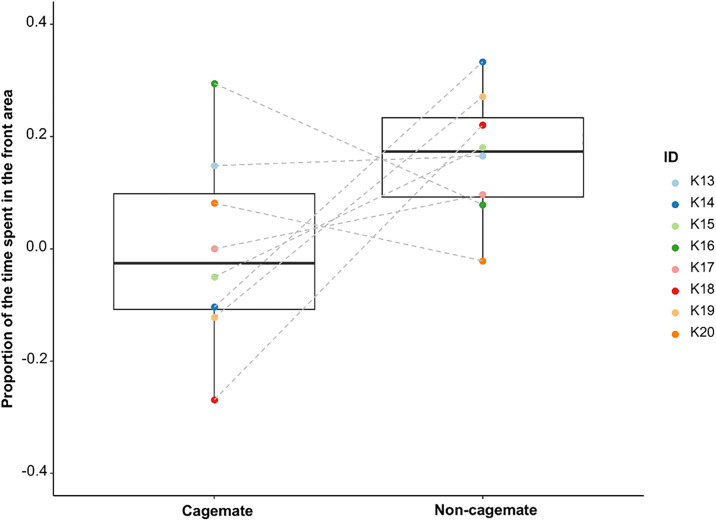
Boxplots for the proportion of the time spent in the front area (N = 8). The vertical axis shows differences in the proportion of time spent between the habituation and test phases.

The three-chamber test revealed that most subjects spent more time in front of the non-cagemate with whom they performed the joint task than with their cagemate (exact Wilcoxon signed-rank test: N = 8, W = 13, p = 0.050, r = 0.570; Fig 4). Therefore, we confirmed that the subjects distinguished between cagemates and non-cagemates and that cagemates were more familiar.

## Discussion

The rats from the two strains showed a joint Simon effect; that is, a larger compatibility effect was observed in the joint task than in the control or paired control tasks. Consistent with previous findings [28], the effect occurred when the partner performed the task but not when the two rats were merely next to each other. This finding suggests that the partner’s actions affected the performance of the other rat. The preliminary examination demonstrated that the size of the joint Simon effect differed depending on the relationship between partners that shared the task, with a larger effect observed when the task was performed with a cagemate than with a non-cagemate.

### Interpretation of the joint Simon effect

The partner of the actor in the paired control was able to press the lever during the test session. As in a previous study [28], the partner first responded to the stimulus in both the cagemate and non-cagemate conditions; however, these responses stopped gradually owing to a lack of contingency for the reward. Nevertheless, the partner’s action did not completely disappear; they occasionally showed lever-pressing behavior, irrespective of the stimulus. A human study revealed that salient actions, such as that by the Japanese waving cat, are sufficient to act as a reference and cause the joint Simon effect [41]. Our results suggest that these occasional actions were not salient enough for the occurrence of the joint Simon effect or that the paired control task was not conjoint between two individuals. However, this possibility cannot be verified solely based on the current study. Therefore, the task for the actor’s partner in the paired control condition should be improved using a lever-pressing task that is not temporally coordinated with the actor [33].

The exploratory test showed that compatibility effects differed depending on familiarity among the dyads in the present study. This indicates the possibility that the representation of a partner’s actions affected the actions of the rats and that performance in incompatible trials decreased in cagemate partners. Two explanations exist for the joint Simon effect in humans: referential coding account [11] and representation of a partner’s action plan for collaborative joint tasks (co-representation) [7]. We did not aim to determine the mechanism of the effects in this study; however, there is no persuasive evidence to support the co-representation of action in rats. Therefore, we adopted a more conservative interpretation to interpret our data: referential coding. According to the referential coding account, spatial information is used to discriminate between the actions of the self and those of others when other cues (action and appearance) are similar. Actual or perceived similarity also increases the effect in human studies. This increase can be modified by manipulating the degree to which animacy is experienced by the partner [42]. Assuming this mechanism, the following hypothesis is proposed: perceived similarity with cagemates increases during the co-housing period and causes a greater effect in cagemate dyads. This hypothesis should be evaluated in future studies with larger sample sizes and balanced subjects in terms of strain and co-housing conditions.

A previous study explored the effects of social relationships on the size of the joint Simon effect in non-human primates; however, no significant social factors were found [29]. One possible explanation for this result is that all subjects were socially tolerant dyads with little variation in their social relationships. This explanation is not inconsistent with our preliminary results, in which subject dyads differed in familiarity. In primates, dyads with less tolerant (even if in the same group) relationships do not easily perform coordination tasks side-by-side and share a reward [43]. This task can be performed in rats, likely because they are relatively tolerant of unfamiliar individuals.

A small joint Simon effect in the non-cagemate condition could be attributed to the decrease in task performance caused by unfamiliarity with the partner. For example, their motivation for the task may have been reduced by curiosity or fear of their non-cagemate partners, consequently affecting their overall performance. However, this possibility is low because the correct rate did not differ between the joint or paired control tasks of the cagemates and non-cagemates. Although the subjects showed interest in non-cagemates placed in the next compartment before the test sessions and showed a preference for them in the three-chamber test, this tendency did not have a significant effect on their performance during the task.

### Validity of the experimental procedure

One difference between our study and previous studies was the reward in the no-go trials. This was adapted for the current study to increase the correct rate of the control or paired control task, such as in the single or joint Simon task, and to achieve a more equal reward condition among the tasks. No compatibility effect would occur if the subjects responded to all no-go tasks in the control or paired control task. Thus, a comparison of the joint and control or paired control conditions would be illogical. However, our subjects showed a certain rate of correct responses in the no-go trials. Consequently, the compatibility effect varied in the no-go trials.

Non-cagemate partners successfully performed the task by ignoring the stimulus for which the partner was in charge, and as a result, the correct rate in the incompatible trials was higher in the non-cagemate condition than in the cagemate condition. Although the compatibility effects differed between the joint task of the cagemate and non-cagemate, the correct rate did not statistically differ between the two conditions. These results reflected that there were individual differences in the responses in the non-cagemate conditions, although most subjects tended to show higher overall correct rates in the non-cagemate than in the cagemate conditions; some non-cagemate pairs showed lower correct rates for both incompatible and compatible trials of the joint task, and the overall correct rates were canceled out. The fact that incorrect go responses did not necessarily increase in the joint Simon task indicates that the corresponding effect is not solely explained by social facilitation.

Correct response rates were lower for the joint, control, and paired control tasks than for the single task. The overall performance on these three tasks decreased mainly because of the incorrect go response in the no-go trials, which was not applicable in the single task. In our experiment, correct no-go responses were rewarded to increase the correct rates in the no-go trials; however, the correct rates did not reach the same level as the single task. This should be improved by inserting training procedures, as previously reported [44].

Although reaction time is generally used to examine the Simon effect in humans, we used the correct response rate as a measure of compatibility effects, which has been used in several non-human studies [26]. This was mainly due to the difficulty in controlling the initial position of the rats in our study, which may have affected the lever-pressing latency. A possible limitation of using accuracy to measure the joint Simon effect in non-human animals is their tendency to exhibit go responses in virtually every no-go trial. Thus, control and paired control tasks do not serve as a control condition [44]. In the current study, this tendency was weaker than that reported in primate studies because of the reward for correct no-go responses. In addition, the compatibility effect was more clearly indicated in error rates than in reaction time in the Simon effect in rats, probably because they have more difficulty suppressing responses to incompatible trials than humans [21]. Nevertheless, whether correct response rates are appropriate measures would be affected by the trade-off between the accuracy and speed of the task [45]. Therefore, a combination of reaction time and correct rates may warrant consideration.

The subjects acted as both cagemate and non-cagemate partners in the joint and paired control tasks. All subjects participated in the test multiple times, and this condition was equal among subjects. In addition, the partner ID was entered in the second model as a random effect. Nevertheless, we cannot rule out the possibility that a specific individual may have caused bias in the results. For example, individual K17 had the lowest correct rates for a single task and for joint tasks with non-cagemate partners. However, the pairs that included K17 did not show the lowest performance among the non-cagemate or cagemate pairs. Moreover, the magnitude of the compatibility effects did not simply reflect the overall correct response rates. Therefore, the effects are unlikely to be explained solely by the performance of a single subject.

We used a relatively similar range of auditory stimuli (2 and 4 kHz) to control the salience for the subjects. However, the stimulus condition had an effect on the first model and a marginal effect on the second. Although we could not determine the reason for the stimulus differences, the salience of the stimuli or lateral bias (the combination of stimulus type and positions was the same for all subjects) may have affected the results. Future studies should address these possibilities.

### Possible effect of social relationship

The subjects were co-housed for at least one month before the first session of the joint task, and familiar individuals were distinguished in the three-chamber test. Therefore, the difference between cagemates and non-cagemates was likely caused by familiarity. A previous helping experiment showed that rats rescued trapped unfamiliar rats of familiar strains [46]. In addition, familiarity with a partner had no effect on emotional contagion or social learning between partners [31,32,47] and cooperation [33]. In contrast, many studies have reported that the behavior of rats varies depending on their familiarity with a particular individual. For example, conditioned fear is greater in rats paired with cagemates than with non-cagemates [48–51], indicating that rats that live together establish emotional and affiliative relationships. These studies indicated that even if an emotional relationship has been formed, certain behaviors are not differentiated by social relationships in terms of their behavior. Our experiment was unlikely to elicit an emotional response. In addition, our setup was not explicitly cooperative. Rather, the rats had to ignore each other and minimize the joint Simon effect to obtain the maximum reward. Our results suggest the possibility that familiarity affects attentional processing through perceived similarity, even in emotionally neutral situations.

## Conclusion

Our study demonstrates a joint Simon effect, indicating that rats cannot ignore adjacent partners during joint tasks, even when they receive no explicit benefit. Our preliminary results on the effect of familiarity suggest the hypothesis that perceived similarity induces the joint Simon effect in rats based on the assumption of referential coding. This hypothesis should be verified with larger sample sizes and balanced subjects to examine how social relationships affect cognitive processing during joint actions in rats.

## Supporting information

S1 Fig Illustration of the front area (180 × 230 mm). The total time spent in each area was used for analysis.
(TIF)

S1 Table Number of sessions used for the statistical analyses of cagemate conditions. (TIF)

S2 Table Multiple comparisons of the compatibility effect of the four task conditions. Significant pairs are shown in bold.
(TIF)

S3 Table Number of trials used for the statistical analyses of non-cagemate conditions. (TIF)

S4 Table Estimated marginal means (EMM) for the interaction effect between task and familiarity conditions. Significant variables are indicated in boldface.
(TIF)

S1 File Datasets. (XLSX)

S2 File R code script. (TXT)
