# Supplementary figures and images for: Joint Simon effect in rats: Validation across two strains and exploratory analysis of the influence of familiarity
Source: PLoS One. 2025 Aug 18;20(8):e0328527. doi: 10.1371/journal.pone.0328527 (PMC12360587; doi:10.1371/journal.pone.0328527)

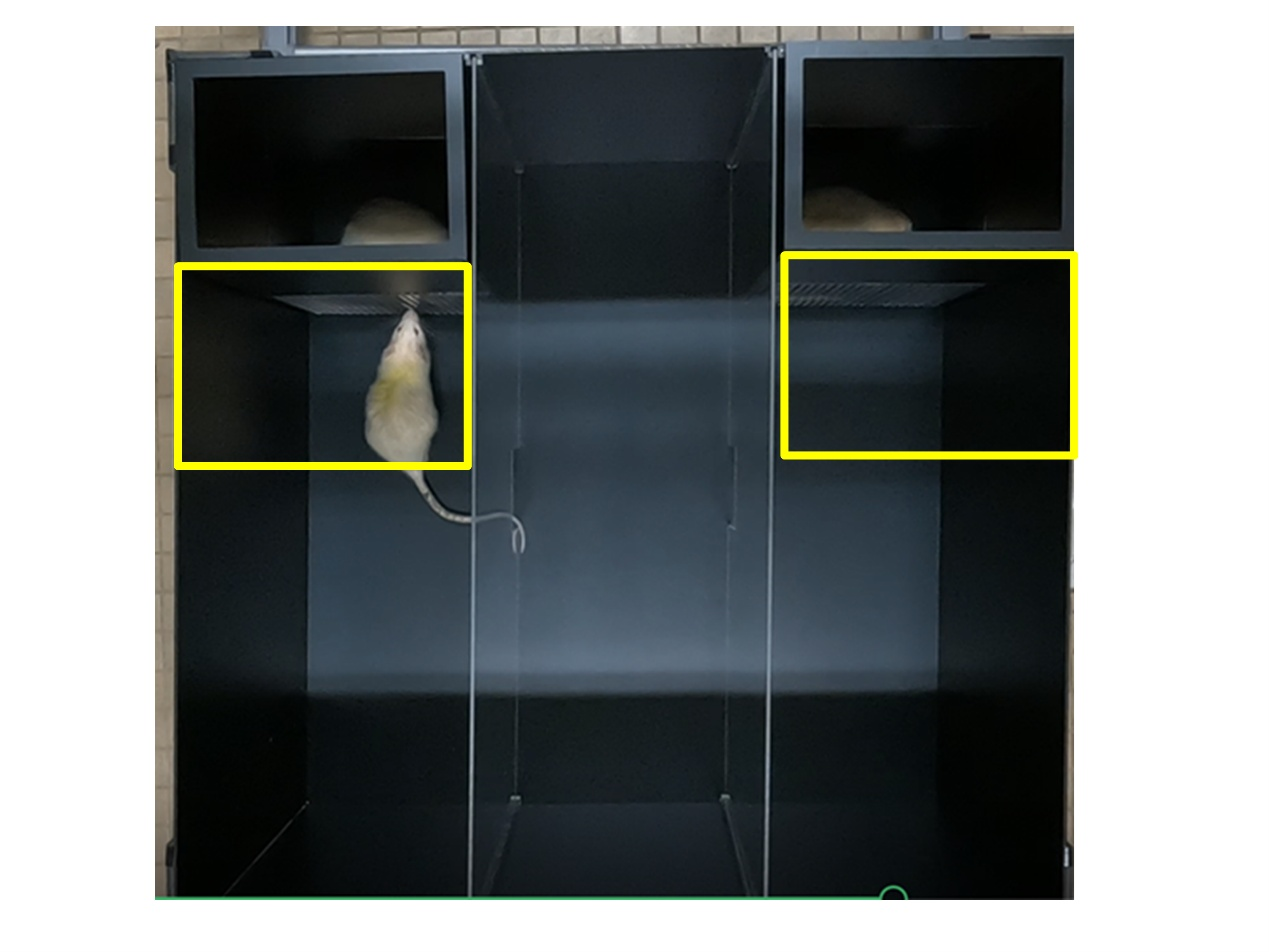

Supplement: S1 Fig — Illustration of the front area (180 × 230 mm). The total time spent in each area was used for analysis. (TIF) [file pone.0328527.s001.tif]

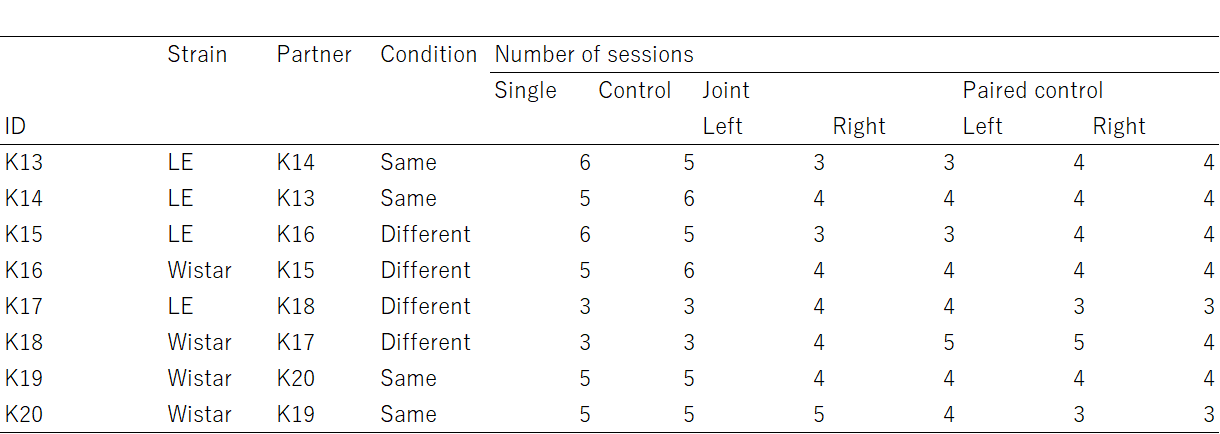

Supplement: S1 Table — (TIF) [file pone.0328527.s002.tif]

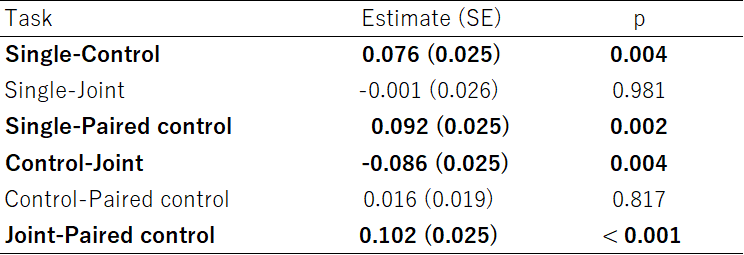

Supplement: S2 Table — Significant pairs are shown in bold. (TIF) [file pone.0328527.s003.tif]

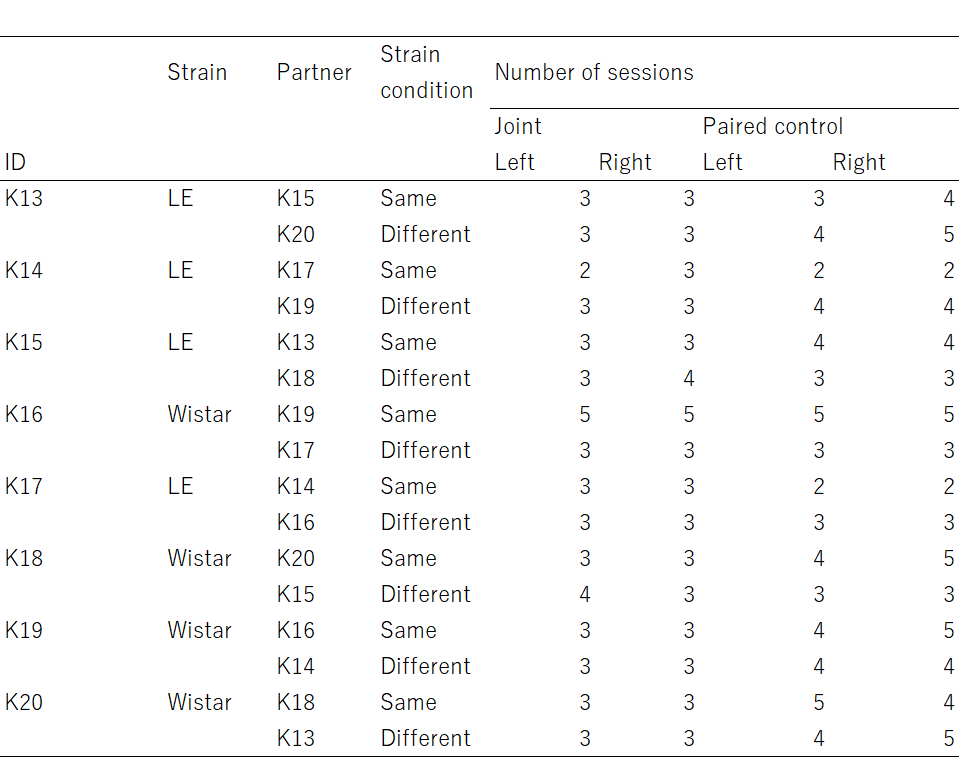

Supplement: S3 Table — (TIF) [file pone.0328527.s004.tif]

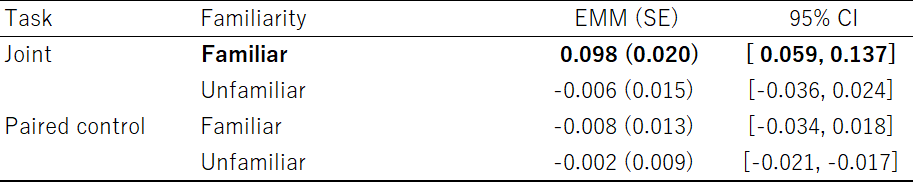

Supplement: S4 Table — Significant variables are indicated in boldface. (TIF) [file pone.0328527.s005.tif]
